# Use of Simethicone After Capsule Ingestion and Its Impact on the Quality of Small Bowel Video Capsule Endoscopy: A Pilot Study

**DOI:** 10.7759/cureus.35307

**Published:** 2023-02-22

**Authors:** Spyridon Zouridis, Gurjiwan Virk, Asra Batool

**Affiliations:** 1 Medicine, Albany Medical Center, Albany, USA; 2 Gastroenterology, Albany Medical Center, Albany, USA

**Keywords:** sbvq, small bowel, polyethylene glycol, simethicone, sbce

## Abstract

Background

Small bowel capsule endoscopy is a tool to visualize the small bowel (SB) for conditions such as obscure bleeding. Various studies have been performed to compare various bowel preparation regimens in terms of small bowel transit time (SBTT), small bowel visualization quality (SBVQ), and diagnostic yield (DY). Literature suggests that using polyethylene glycol (PEG) prep is significantly better compared to clear liquid and overnight fast in terms of SBVQ and DY. Other investigators have tried to assess the efficacy of adding simethicone to the bowel preparation regimen which seems to improve SBVQ. However, no studies have been done to assess the results of simethicone ingestion after capsule swallowing. We intend to give patients simethicone one hour after capsule ingestion for two consecutive hours and compare results for SBVQ pre-and post-ingestion groups. The objective of this study is to compare the effect of simethicone on SBVQ in pre- and post-capsule ingestion groups.

Methodology

This prospective, randomized controlled trial included patients who were scheduled for outpatient capsule endoscopy at Albany Medical Center (AMC) Endoscopy Suite. Patients were divided into the control group, group 1, and the treatment group (group 2). The control group followed the standard AMC pre-capsule protocol that included PEG 238 g the evening prior. Group 1 included patients who received 3 mL of simethicone (20 mg/0.3 mL) 20 minutes prior to ingesting the capsule. The treatment group (group 2) included patients who ingested simethicone 3 mL 20 minutes prior to capsule swallowing, 3 mL after one hour, and 1.5 mL after another hour, totaling 7.5 mL of simethicone. Data regarding SBVQ for every patient were evaluated as an individual zone score from 1-3 points, each in proximal, middle, and distal SB based on the SBTT. A cumulative score of 3-9 was given after adding the three zones. These scores were derived using the Boston Bowel Preparation Scale. Data analysis was done using Microsoft Excel software.

Results

There were six patients in the control group, eight in group 1, and eight in the treatment group (group 2). Proximal, middle, and distal SB did not show any significant difference between their SBVQ scores. Moreover, the total combined score also showed no statistical difference in the SBVQ score.

Conclusions

There were no statistically significant differences in the SBVQ neither while looking at the cumulative score nor individual segmental score of the entire SB. However, this is only a pilot project with a small number of subjects and results may differ in future studies with increased power.

## Introduction

Small bowel capsule endoscopy (SBCE) was first introduced in clinical practice in the early 2000s and since then has been widely used to visualize the small bowel (SB) for diagnostic purposes [1,2]. SBCE is utilized for the investigation of small bowel bleeding, inflammatory bowel disease, polyposis syndromes, suspected SB neoplasia, and celiac disease [2,3]. Multiple studies have been performed in the past to compare various SBCE bowel preparations, mainly in terms of small bowel transit time (SBTT), small bowel visualization quality (SBVQ), and diagnostic yield (DY). Most investigators have identified that the best bowel preparation regimen is the use of purgative agents and, more specifically, polyethylene glycol (PEG). Anti-foaming agents, such as simethicone, have also been studied, but not extensively. Literature reveals improved SBVQ when simethicone is added to the regimen [4]. Even though the bowel prep administration timing is significant for better mucosal visualization, simethicone administration timing has not been studied. The current study intends to investigate this by assessing SBVQ when simethicone is administered at different times including administration after capsule swallowing [3].

This was submitted but not presented at the American College of Gastroenterology 2018 conference. Since then, more patients were enrolled in the study.

## Materials and methods

We conducted a prospective, randomized controlled study in patients who were scheduled for outpatient SBCE at Albany Medical Center (AMC). The patients were divided into three groups, namely, control, group 1, and treatment (group 2) groups. The control group followed the standard AMC pre-capsule protocol that included PEG 238 g the evening prior. Group 1 included patients who received 3 mL of simethicone 20 mg/0.3 mL 20 minutes prior to ingesting the capsule. The treatment group included patients who had simethicone 3 mL (20 mg/0.3 mL) 20 minutes prior to capsule swallowing, 3 mL after one hour, and an additional 1.5 mL after another hour. Group 2 (treatment group) was offered two additional doses of simethicone after capsule endoscopy to assess the effect of simethicone on preventing bubbles as they form during capsule passage. The control group, Group 1, and treatment group included six, eight, and eight patients, respectively.

The Boston Bowel Preparation Scale system was used to analyze the SBVQ [5]. It was divided into proximal, middle, and distal SB based on the SBTT. Scores ranging from 1 to 3 were assigned to each segment with a cumulative score of 3-9. The physician reading the capsule study was blinded to the patient group.

## Results

**Figure 1:**
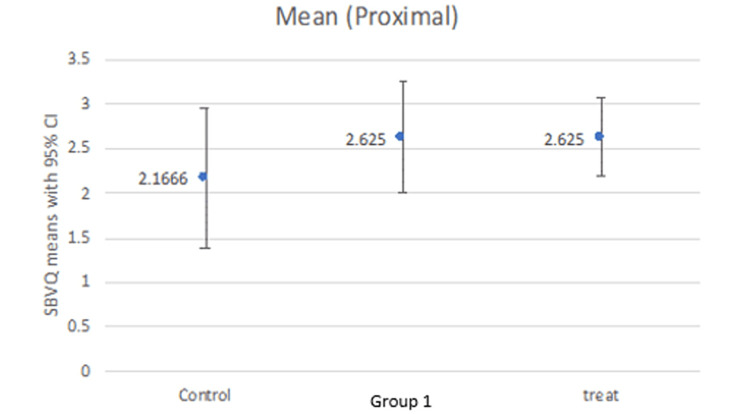
Small bowel visualization quality scores in the proximal zone.

**Figure 2:**
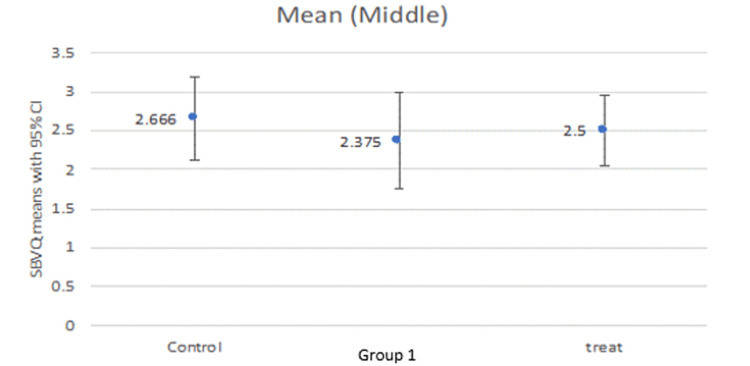
Small bowel visualization quality scores in the middle zone.

**Figure 3:**
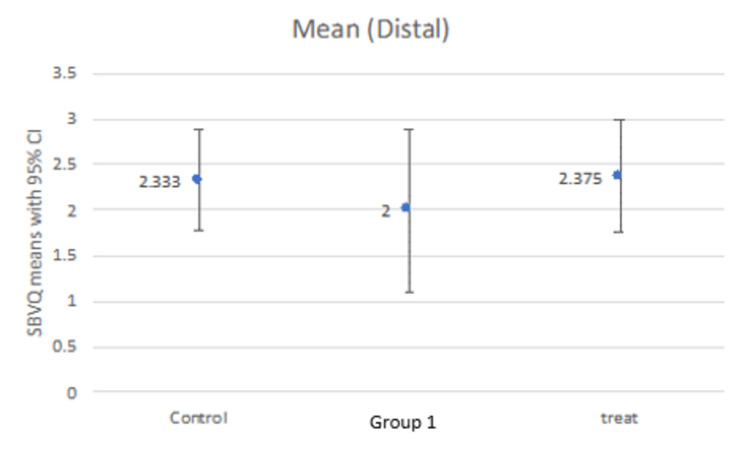
Small bowel visualization quality scores in the distal zone.

**Figure 4:**
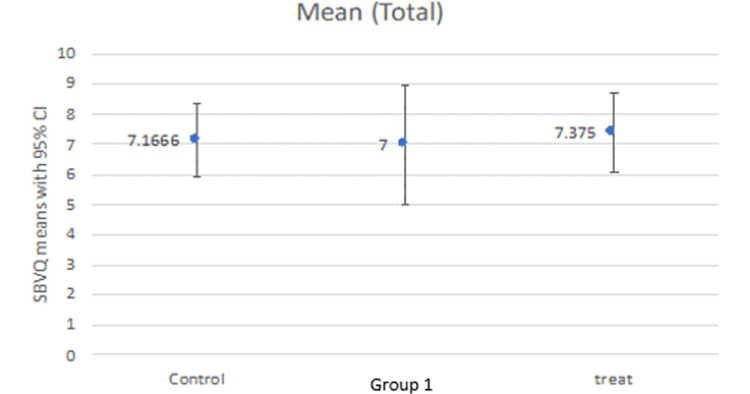
Cumulative small bowel visualization quality scores.

Our study compared the SBVQ score in every individual zone (proximal, middle, distal) for all groups as well as the total score. The mean score in the proximal zone was 2.16, 2.62, and 2.625 for the control group, group 1, and treatment group, respectively (Figure 1). The middle zone mean score was 2.66, 2.37, and 2.50 for the control group, group 1, and treatment group, respectively (Figure 2), while the distal zone mean score was 2.33, 2.00, and 2.37, respectively (Figure 3). After calculating the scores in individual zones and adding them, the total mean SBVQ score was 7.16, 7, and 7.37 for the control group, group 1, and treatment group, respectively. These minor variations had no statistically significant difference (Figure 4).

## Discussion

SBTT plays a significant role in the accuracy of SBCE results. Prolonged SBTT leads to increased DY, while it has been revealed that approximately 220 minutes is adequate transit time to identify bleeding lesions when SBCE is used to investigate obscure gastrointestinal bleeding [6,7]. Studies have revealed that various purgative agents and simethicone used in SBCE do not alter the SBTT [8-10].

Extensive investigation has been conducted on the use and efficacy of bowel preparation regimens prior to SBCE. Most studies have compared various purgative agents as opposed to fasting or other purgative medications, reporting results in terms of SBVQ and DY [3,11-13]. PEG, a non-absorbable polymer that creates a significant osmotic effect, when used prior to SBCE performed better not only when compared to fasting but also when compared to other solutions in terms of both SBVQ and DY; however, there can be some remaining bubbles that can lower the SBVQ [11-14].

Antifoaming agents such as simethicone have also been suggested as part of the bowel preparation regimen. Hypothetically, simethicone prevents bubble formation and/or decreases the surface tension of air bubbles leading to coalescing into larger bubbles that pass through flatulence [4,15]. Indeed, the use of antifoaming agents along with PEG prior to SBCE showed a decrease in the amount of bubble gas seen during SBCE and improved SBVQ when compared to fasting alone [4,9,15]. Simethicone has also been used as single-agent bowel preparation prior to SBCE, and even though it led to improved SBVQ when compared to fasting alone, purgative agents had better outcomes [16]. The contribution of simethicone to DY increase is highly debatable [4].

The timing of bowel preparation administration has also been discussed in previous studies. The administration of purgative agent solution shortly prior to capsule ingestion seems to significantly improve SBVQ, and, in fact, the relationship between the time of completion of bowel preparation till capsule ingestion and SBVQ is inversely proportional [3]. The hypothesis underlying this observation is that the removal of bile, fluids, and other bowel content remains better when the bowel preparation is given closer to capsule ingestion. Even though bowel preparation timing as described earlier improved SBVQ, this did not clearly benefit DY [3].

Even though the timing of purgative agent administration has been investigated in the past, this does not apply to simethicone. This is the first study to investigate the timing of simethicone administration and whether this plays a role in its effectiveness. The investigation led to insignificant differences in terms of SBVQ in the study groups when simethicone was administered at different times. The mean SBVQ assigned by the reading gastroenterologist in the proximal, middle, and distal SB zones (as defined by SBTT) was not significantly different between the control group, group 1, and treatment group. As a result, the total SBVQ score was also not significantly different between the groups. Interestingly, despite prior studies showing better outcomes when purgative agents are combined with simethicone, this study did not observe any significant differences between the control group where no simethicone was offered, and the other two groups that received the antifoaming agent.

Our study is limited due to the small number of subjects in each group. Additionally, the study could not be conducted further due to joint commission rules on the storage of simethicone. Despite the fact that studies in the past have revealed SBVQ improvement by simethicone use, this study did not have similar results, most probably due to inadequate power. As such, studies with more subjects should be conducted to elucidate the effect of simethicone on bowel preparation for SBCE when different administration timing is used.

## Conclusions

PEG use consistently performs better as an SBCE bowel preparation agent in terms of both SBVQ and DY without affecting the SBTT. The addition of simethicone on a purgative agent bowel preparation regimen leads to enhanced SBVQ by reducing the amount of air bubbles present in the bowel. Administering purgative agents, closer to the capsule swallowing time, is also helpful and increases SBVQ. However, based on the results of this study, administration of simethicone at different times prior to capsule ingestion does not affect the SBVQ. Moreover, this study challenges prior observations supporting SBVQ improvement with a combination of purgative agents and antifoaming medications. Despite these results, the current study is limited by the small number of subjects.
